# Complete plastome sequence of *Torenia concolor* Lindley (Linderniaceae): an ornamental herb

**DOI:** 10.1080/23802359.2019.1627949

**Published:** 2019-07-11

**Authors:** Xia-Lan Cheng, Hai-Li Li, Hong-Xin Wang, Mir Mohammad Nizamani, Yan Chen

**Affiliations:** a Lingnan Normal University, Guangdong, Zhanjiang, China;; b Institute Arts, Sanya University, Hainan, Sanya, China;; c Hainan Key Laboratory for Sustainable Utilization of Tropical Bioresources, Hainan University, Haikou, China

**Keywords:** *Torenia concolor* Lindley, plastome, phylogeny, genome structure, Linderniaceae

## Abstract

In this study we firstly reported the complete chloroplast genome of *Torenia concolor*, a species of Linderniaceae. The complete chloroplast genome of *T. concolor* is 153,853 bp in length with a typical quadripartite structure, consisting of a large single-copy region (LSC, 85,446 bp), a single-copy region (SSC, 18,837 bp), and a pair of inverted repeats (IRs, 24,785 bp). There are 114 genes annotated, including 80 unique protein-coding genes, 4 unique ribosomal RNA genes, and 30 transfer RNA genes. To investigate the evolution status of *T. concolor*, as well as Linderniaceae, we constructed a phylogenetic tree with *T. concolor* and other 16 species based on their complete chloroplast genomes. According to the phylogenetic topologies, *T. concolor* was closely related to *Pedicularis hallaisanensis*.

*Torenia concolor* Lindl. belongs to family Linderniaceae. It is a creeping herb with showy blue-purple flowers. The distribution of *T. concolor* were in southern provinces of China, including Guangdong, Guangxi, Guizhou, Hainan, Taiwan, Yunnan, and Laos, Vietnam, Ryukyu Islands of Japan (China ECoFo, 2013). *Torenia concolor* could be used as ornamental plants due to its highly aesthetic value, and it is also a traditional Chinese medicinal herb widely used for the treatment of aching muscles and bones, heat stroke, dysentery, cold, and ambustion (Liang et al. 2017).

As chloroplast carry maternal genes, it is important in phylogeny reconstruction. However, to date, there have been no published plastome sequences for *T. concolor* chloroplast. The genetic and genomic information is urgently needed to promote its systematics research and develop molecular markers to improve its aesthetic trait further. Here, we report and characterize the complete plastid genome sequence of *T. concolor* (GenBank accession number: MK789685) in order to provide genomic resources useful for promoting its conservation and garden utilization.

In this study, the fresh leaves of *T. concolor* were collected from its natural habitat Xinfeng village, Gaozhou county, China (E111°11′27.49″ N22°01′10.35″), with the voucher specimens (LNH180702005) deposited in the Herbarium of Lingnan Normal University, Zhanjiang, China. The experiment procedure was as reported in Gao et al. (2017). Around 6 Gb clean data were assembled against the plastome of *T. asiaticum* (MG963252.1) using MITO bim V1.8 (Hahn et al. 2013). The plastome was annotated using Geneious R8.0.2 (Biomatters Ltd., Auckland, New Zealand) against the plastome of *T. asiaticum* (MG963252.1). The annotation was corrected with DOGMA (Wyman et al. 2004).

The plastome of *T. concolor* was found to possess a total length 153,853 bp with the typical quadripartite structure of angiosperms, containing two inverted repeats (IRs) of 24,785 bp, a large single-copy (LSC) region of 85,446 bp and a small single-copy (SSC) region of 18,837 bp. The plastome contains 114 genes, consisting of 80 unique protein-coding genes, 30 unique tRNA genes, and 4 unique rRNA genes. The overall A/T content in the plastome of *T. concolor* is 62.40%, for which the corresponding value of the LSC, SSC, and IR regions were 64.60%, 68.00%, and 56.50%, respectively.

We used RAxML (Stamatakis 2006) with 1000 bootstraps under the GTRGAMMAI substitution model to reconstruct a maximum likelihood (ML) phylogeny of *T. concolor* and 16 published complete plastomes of Scrophulariaceae, Plantaginaceae, Orobanchaceae, and Gesneriaceae, using *Lysionotus pauciflorus* and *Streptocarpus teitensis* (Gesneriaceae) as outgroups. According to the phylogenetic topologies, *T. concolor* was closely related to *Pedicularis hallaisanensis*. Most nodes in the plastome ML trees were strongly supported (Figure 1). The complete plastome sequence of *T. concolor* provides genetic data critical for the study of evolution and systematics of *Torenia* and garden utilization for itself.

**Figure 1.**
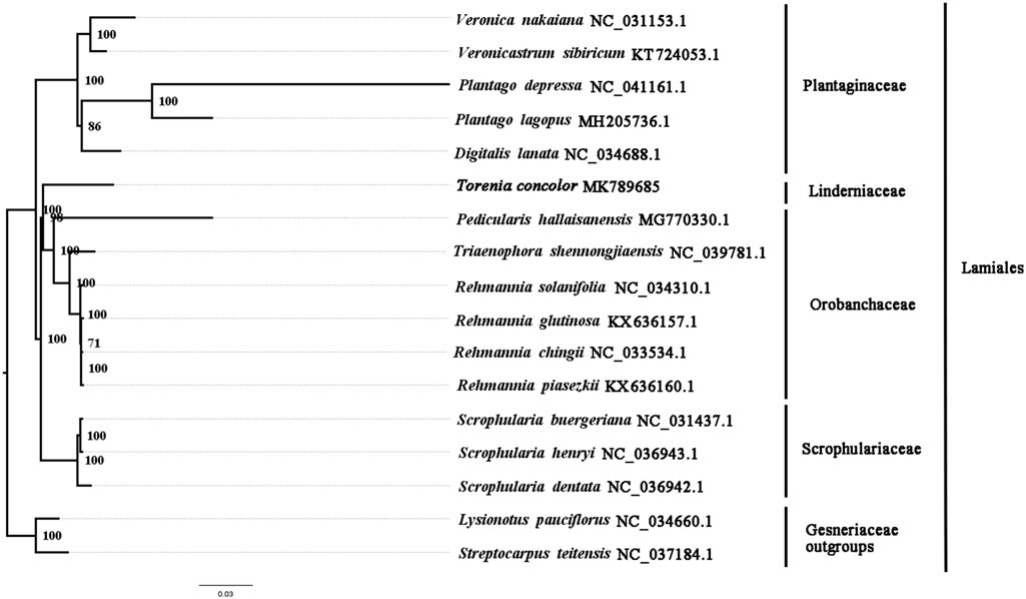
ML phylogenetic tree of *T. concolor* with 17 species was constructed by chloroplast genome sequences. Numbers on the nodes are bootstrap values from 1000 replicates. *Lysionotus pauciflorus* and *Streptocarpus teitensis* were selected as outgroups.
